# *dmrt2* and *myf5* Link Early Somitogenesis to Left-Right Axis Determination in *Xenopus laevis*

**DOI:** 10.3389/fcell.2022.858272

**Published:** 2022-06-23

**Authors:** Melanie Tingler, Amelie Brugger, Kerstin Feistel, Axel Schweickert

**Affiliations:** Department of Zoology, Institute of Biology, University of Hohenheim, Stuttgart, Germany

**Keywords:** left-right asymmetry, *dmrt2*, *myf5*, somitogenesis, cilia, paraxial patterning, *Xenopus*, embryo

## Abstract

The vertebrate left-right axis is specified during neurulation by events occurring in a transient ciliated epithelium termed left-right organizer (LRO), which is made up of two distinct cell types. In the axial midline, central LRO (cLRO) cells project motile monocilia and generate a leftward fluid flow, which represents the mechanism of symmetry breakage. This directional fluid flow is perceived by laterally positioned sensory LRO (sLRO) cells, which harbor non-motile cilia. In sLRO cells on the left side, flow-induced signaling triggers post-transcriptional repression of the multi-pathway antagonist *dand5*. Subsequently, the co-expressed Tgf-*β* growth factor Nodal1 is released from Dand5-mediated repression to induce left-sided gene expression. Interestingly, *Xenopus* sLRO cells have somitic fate, suggesting a connection between LR determination and somitogenesis. Here, we show that doublesex and mab3-related transcription factor 2 (Dmrt2), known to be involved in vertebrate somitogenesis, is required for LRO ciliogenesis and sLRO specification. In *dmrt2* morphants, misexpression of the myogenic transcription factors *tbx6* and *myf5* at early gastrula stages preceded the misspecification of sLRO cells at neurula stages. *myf5* morphant tadpoles also showed LR defects due to a failure of sLRO development. The gain of *myf5* function reintroduced sLRO cells in *dmrt2* morphants, demonstrating that paraxial patterning and somitogenesis are functionally linked to LR axis formation in *Xenopus*.

## Introduction

Organ asymmetry is present in all animal phyla. In vertebrates, left-right (LR) asymmetry is determined after the dorsal-ventral and anterior-posterior body axis have been established during gastrulation. The mechanism of symmetry breakage depends on the leftward movement of extracellular fluid during neurula stages. This flow is generated by a transient mono-ciliated epithelium in the embryonic midline of the archenteron, referred to as the left-right organizer (LRO). LROs are highly conserved, and are found in most vertebrates and probably also in other deuterostome species (Blum et al., 2009; Tisler et al., 2016; Blum and Ott, 2018; Zhu et al., 2019; Little and Norris, 2020). LROs are characterized by the subdivision into two distinct cell types: flow-generating and flow-sensing cells. Centrally localized LRO (cLRO) cells harbor motile cilia, whereas bilaterally flanking sensory LRO (sLRO) cells project non-motile cilia. Importantly, only sLRO cells express the Dand5/Nodal/Gdf3 module, which is the molecular target of flow-triggered signal transduction. In the absence of flow, the secreted Cerberus type inhibitor Dand5 complexes with the Tgf-β morphogen Nodal and the Tgf-*β* growth factor Gdf3 (Gdf1 in mice), thereby preventing Nodal/Gdf3 heterodimers from spreading and interacting with their cognate receptor (Vonica and Brivanlou, 2007; Nakamura et al., 2012; Pelliccia et al., 2017). After flow detection, Dand5 levels decrease in left sLRO cells and consequently, Nodal/Gdf3 is freed from repression (Hojo et al., 2007; Schweickert et al., 2010; Blum and Ott, 2018; Little and Norris, 2020). Recently, we and others demonstrated that Dand5 reduction is due to the inhibition of *dand5* mRNA translation and its subsequent decay. In these studies, the RNA binding protein Bicaudal C1 was identified as the post-transcriptional mediator of flow-induced signaling leading to *dand5* mRNA repression (Maerker et al., 2021; Minegishi et al., 2021). Upon Dand5 reduction, Nodal is released from sLRO cells and conveys left positional information to the left lateral plate mesoderm (LPM). In left LPM cells, Nodal signaling induces three direct target genes: *nodal* itself, the secreted Nodal feedback inhibitor *lefty,* and the homeobox transcription factor *pitx2*, which together constitute the so-called Nodal cascade. Unlike *nodal* and *lefty*, which are only expressed during a short time window, left-sided *pitx2* expression is maintained in the LPM and is thought to govern asymmetric organogenesis (Campione et al., 1999; Tanaka et al., 2007; Grimes and Burdine, 2017).

Before the onset of gastrulation, LRO precursor cells are specified on the outside of the embryo and are subsequently internalized by the tissue movements of gastrulation. Using cell labeling at blastula and gastrula stages, LRO precursors, i.e., dorsal forerunner cells or superficial mesoderm (SM), were identified in fish and frogs, respectively (Cooper and D’Amico, 1996; Shook et al., 2004; Warga and Kane, 2018). Today, mRNA expression of the forkhead box transcription factor *foxj1*, a master control gene for motile cilia, suffices to detect vertebrate LROs or their precursor cells by whole-mount *in situ* hybridization (WMISH) (Aamar and Dawid, 2008; Zhang et al., 2004; Stubbs et al., 2008; Beyer et al., 2012). In early *Xenopus* gastrulae, SM cells are positioned animally to the Spemann organizer in a crescent-shaped manner (Shook et al., 2004; Blum et al., 2014b). Various signaling pathways impact SM specification including canonical Wnt and Fibroblast growth factor (Fgf) signaling (Glinka et al., 1996; Stubbs et al., 2008; Walentek et al., 2013; Vick et al., 2018; Schneider et al., 2019). Inhibition of Wnt or Fgf signal transduction results in the loss of *foxj1* expression, which affects ciliogenesis and morphogenesis of the cLRO and alters laterality. *foxj1* is required for the motility of cilia on the flow-generating cLRO cells, but it is currently unknown how the specification of sLRO cells bearing non-motile cilia is achieved. In addition, SM labeling or the expression analysis of mesodermal marker genes such as *tbxt* and *myod1* demonstrates differences in cLRO and sLRO fate being notochordal and somitic, respectively (Shook et al., 2004; Schweickert et al., 2010). We and others recently showed that Fgf signaling is crucial for sLRO and presomitic cells, suggesting a tight connection between sLRO morphogenesis and paraxial patterning/somitogenesis (Sempou et al., 2018; Schneider et al., 2019). This notion is substantiated by the requirement of the t-box transcription factor Tbx6 for somitogenesis and LRO morphogenesis in mice (Concepcion et al., 2018). In addition, Dmrt2, a transcription factor of the doublesex and mab3-related family, is crucial for somite development, and its loss-of-function results in LR defects in fish embryos (Meng et al., 1999; Saúde et al., 2005; Liu et al., 2009).

Here, we report that Dmrt2 regulates the formation of cLRO and sLRO cells in *Xenopus laevis*. Dmrt2 was required for *foxj1* expression in the SM and consequently for LRO ciliogenesis. In addition, Dmrt2 was essential for sLRO formation, which was due to a function in paraxial mesodermal patterning. We show that the myogenic transcription factor Myf5 is required for LR development, acting downstream of Dmrt2 on sLRO formation. Our data reveal a direct link between patterning of the paraxial mesoderm and sLRO morphogenesis.

## Results

### Dmrt2 Activity Is Required for LR Axis Development and LRO Ciliogenesis

To understand the relationship between somitogenesis and LR axis formation in the *Xenopus* embryo, *dmrt2* was chosen for analysis because it is expressed in the fish LRO (Kupffer’s vesicle), suggesting a specific role during symmetry breakage (Saúde et al., 2005; Liu et al., 2009; Lourenço et al., 2010). In early tadpole stages, *dmrt2* is expressed in somitic tissue as demonstrated by whole-mount *in situ* hybridization (WMISH; data provided by Soeren S Lienkamp @ Xenbase; Bowes et al., 2009), indicating a conserved activity within vertebrates. Using WMISH, we detected strong *dmrt2* expression in the *Xenopus* LRO at neurula stages, resembling expression in the fish LRO. However, *dmrt2* was restricted to flow-generating cLRO cells, while lateral sLRO cells did not express *dmrt2* (Supplemental Figures S1A, A′).

**FIGURE 1:**
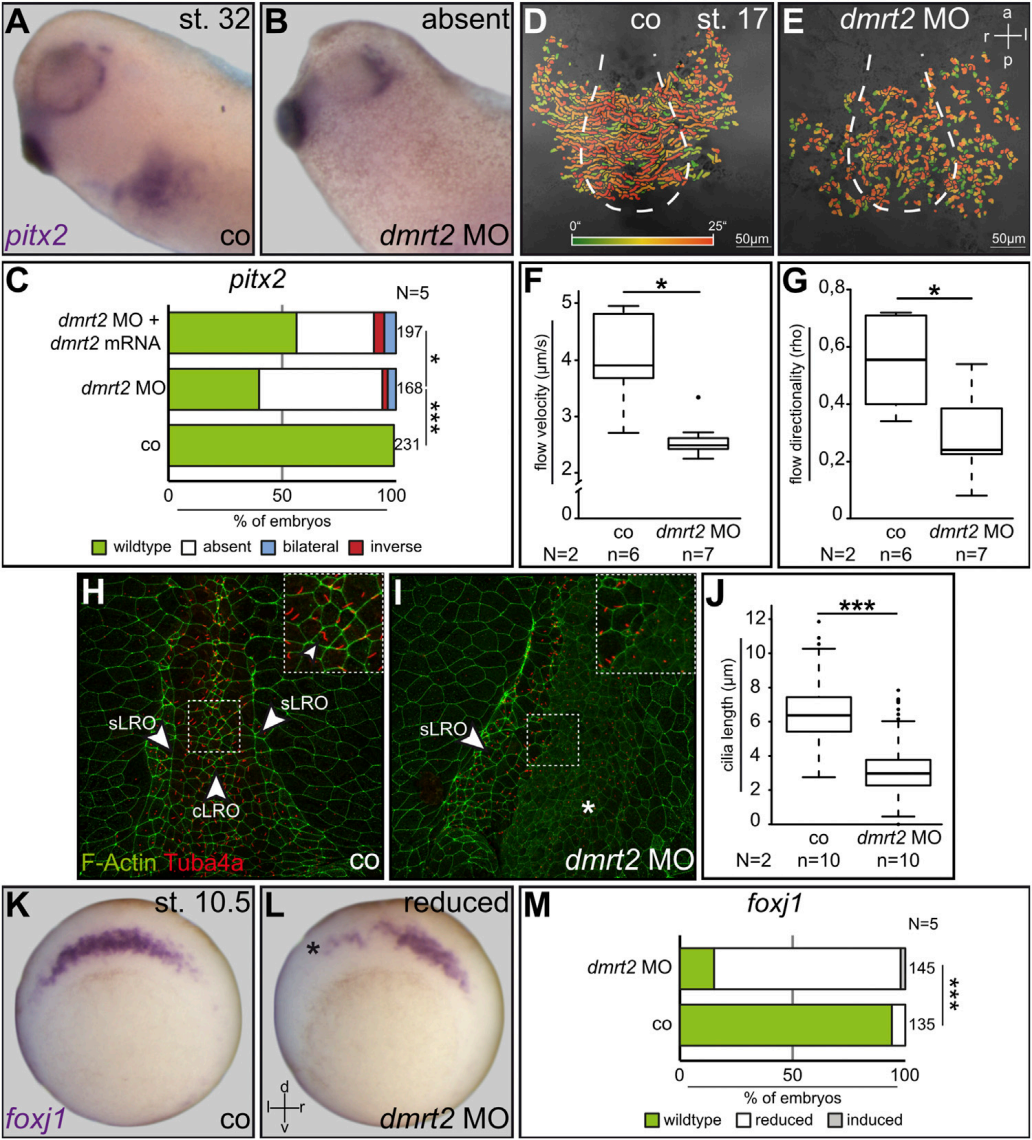
Dmrt2 is required for left-right development. Expression of the LR marker *pitx2* in the left LPM was lost in 60% of specimens after left-sided injection of *dmrt2* MO **(A–C)**. Co-injection of full-length *dmrt2* mRNA statistically significantly restored *pitx2* asymmetry **(C)**. Leftward fluid flow in controls and bilaterally injected *dmrt2* morphants which was perturbed **(D,E)** in velocity **(F)** and directionality **(G)**. LROs of controls and *dmrt2* knockdown embryos were analyzed by immunofluorescence, detecting F-actin (green) and Tuba4a [red; **(H,I)**]. Compared to controls, cilia were statistically significantly shorter in morphants **(G)**. Note enhanced F-actin signals in lateral sensory LRO cells [**(H)**, sLRO] compared to flow-generating central LRO cells [**(H)**, cLRO]. Enhanced F-actin signals were lost on the *dmrt2* MO-injected side **(I)**. SM expression of *foxj1* in wildtype embryos **(K)** was diminished by *dmrt2* loss of function **(L,M)**. Numbers (n) in **(C,F,G,J,M)** represent analyzed specimens. N represents the number of independent experiments. Statistical analyses were done with one-sided Pearson’s chi-square test **(C,J,M)** or Wilcoxon-Match-Pair test **(F,G)**; * significant *p* < 0.05; ***, very highly significant *p* < 0.001. Asterisks mark injected sides; a = anterior; co = control; cLRO = central left-right organizer; l = left; p = posterior; r = right; sLRO = sensory left-right organizer.

A unique feature of the frog system is the ability to restrict experimental manipulations in the early embryo on the left or right side, making it particularly suited to analyze LR axis development. Unilateral injections of synthetic mRNAs or antisense morpholino oligos (MO) into four to eight cell embryos allow to perform site-directed gain- or loss-of-function experiments and analyzing their impact on LR axis formation. To analyze the potential role of Dmrt2 during LR development, a translation-blocking morpholino oligo (*dmrt2* MO) was designed. *dmrt2* MO was injected in a site-specific manner and laterality was determined by *pitx2* expression. Untreated controls and right-sided *dmrt2* knockdown showed wildtype (WT) *pitx2* asymmetry (Figure 1A and not shown). Left *dmrt2* MO injections, however, resulted in the loss of left *pitx2* transcription in about 60% of cases (Figures 1B, C). Importantly, asymmetry was statistically significantly restored by co-injecting full-length *dmrt2* mRNA, which was insensitive to the *dmrt2* MO, indicating the specificity of the observed phenotype (Figure 1C). Next, we analyzed the effect of *dmrt2* loss of function on leftward flow. *dmrt2* MO was bilaterally injected into four to eight cell embryos, targeting the central LRO lineage. The dorsal explants of neurula embryos were dissected and morphants, as well as untreated controls, were processed for flow analysis by adding fluorescent microbeads and subsequent recording of bead motion. While controls showed WT leftward movement of beads (Figures 1D, F, G), flow velocity and directionality were statistically significantly diminished in *dmrt2* morphants (Figures 1E, F, G), demonstrating that Dmrt2 is required for cilia-driven symmetry breakage. Next, flow-generating LRO cilia of controls and unilaterally injected morphants were analyzed by immunofluorescence (IF) using an anti-acetylated tubulin antibody. F-actin staining using fluorescently tagged phalloidin visualized cell borders. WT cLRO cells were ciliated and cilia length was around 6 µm on average (Figures 1H, J), matching our previous findings (Schweickert et al., 2007). Although the pattern of ciliation was unaltered in *dmrt2* morphants (Figure 1I), cilia were substantially shortened to about 2–2.5 µm (Figures 1I, J), providing an explanation for flow deficiency. Recently, the master regulator of motile cilia, *foxj1,* was shown to be a transcriptional target of Dmrt2 in fish (Pinto et al., 2018). The loss of flow and shortened cilia in *dmrt2* morphants could therefore reflect impaired *foxj1* expression in the cLRO precursor cells at gastrula stages. Indeed, 80% of unilaterally injected *dmrt2* morphants showed diminished *foxj1* expression in the SM on the targeted embryo half (Figures 1K–M), which correlated with defective ciliogenesis at the LRO. Next, we asked whether *dmrt2* was differentially expressed in SM and the underlying deep mesoderm (DM). To address this question, dorsal mesodermal explants of early gastrula embryos were dissected and further bisected into SM and DM. Using RT-PCR, *dmrt2* mRNA was detected in both tissues (Supplemental Figures S1B, C). However, *dmrt2* knockdown did not diminish DM expression of the organizer genes *goosecoid* and *chordin* (Supplemental Figures S1D–G), which excludes an impact on organizer formation. We conclude that at gastrula stages, Dmrt2 activity is required for cLRO morphogenesis and thus for correct LR development.

### sLRO Morphogenesis Depends on Dmrt2 Activity

**FIGURE 2:**
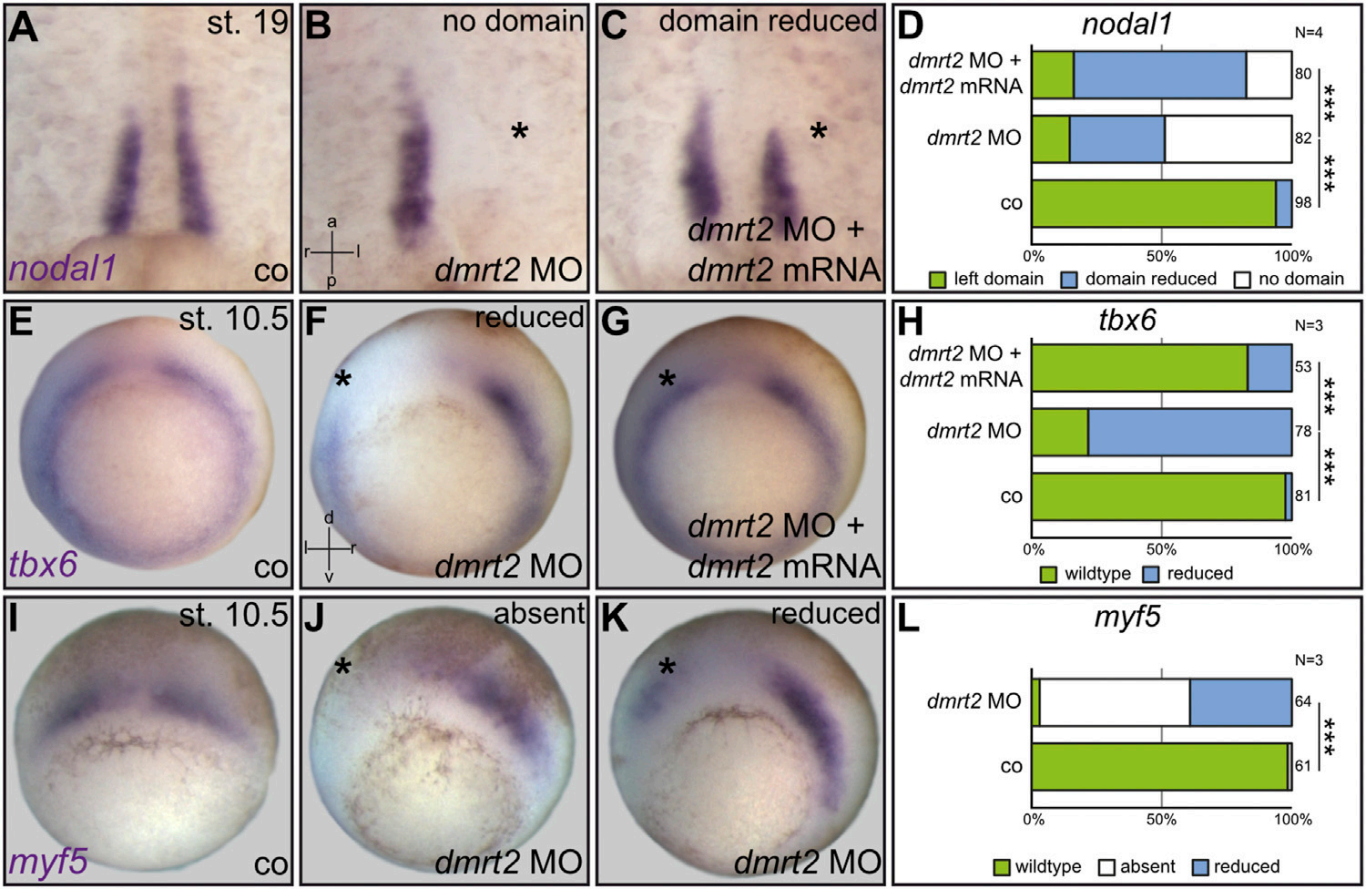
Loss of *nodal1* in sLRO cells by *dmrt2* knockdown correlates with impaired expression of the somitic marker genes *tbx6* and *myf5* at gastrula stages. *nodal1* mRNA was analyzed at stage (st.) 19 in untreated controls [co, **(A)**], unilaterally injected *dmrt2* MO morphants **(B)** or specimens that received a mix of *dmrt2* MO and rescue *dmrt2* mRNA **(C)**. *nodal1* expression was lost or reduced in *dmrt2* morphants **(B,D)**. Statistical analysis shows that *nodal1* was very highly significantly rescued **(C,D)**. Early gastrula embryos were stained for *tbx6* and *myf5* **(E–L)**. Controls showed horseshoe-like *tbx6* expression, omitting the Spemann organizer **(E)**. *tbx6* signals were reduced by *dmrt2* MO on the injected side **(F,H)**, which by statistics was significantly rescued upon co-injection of *dmrt2* mRNA **(G,H)**. The angel wing-like expression pattern of *myf5* **(I)** was lost **(J,L)** or reduced in *dmrt2* morphants as well **(J,L)**. The asterisk mark the injected side. Numbers in **(D,H,L)** represent analyzed specimens. N represents the number of independent experiments. Statistical analyses were done with one-sided Pearson’s chi-square test **(D,H,L)**; very highly significant, *p* < 0.001. a = anterior; co = control; l = left; p = posterior; r = right; d = dorsal; v = ventral.

During the analysis of LRO cilia, we noted that in untreated specimens, F-actin staining was more intense in sLRO than cLRO cells (Figure 1H; data not shown). Enhanced actin signals can be the consequence of apical constriction, a cell shape change observed in sLRO cells (Shook et al., 2004). Surprisingly, this sLRO-specific actin staining was not detected in *dmrt2* morphants (Figure 1I). Based on our recent work on Fgf function during LRO morphogenesis (Schneider et al., 2019), we proposed that either apical constriction failed or sLRO cells were entirely absent. To analyze this sLRO phenotype in more detail, the expression of *nodal1* was assessed. The morphogen *nodal1* is specifically expressed in sLRO cells and is required to transfer left identity to the LPM (Figure 2A; Blum et al., 2014b). Targeting left sLRO cells (c.f. Tingler et al., 2014) with *dmrt2* MO diminished *nodal1* signals (Figures 2B, D), suggesting that this effect contributed to the failure of Nodal cascade induction in the left LPM. Co-injecting full-length *dmrt2* mRNA restored *nodal1* expression in morphants, although domains were generally smaller in size compared to WT embryos (Figure 2C). Similar results were obtained when right-sided knockdown was performed or *dand5* was analyzed (data not shown). In addition, *myod1* expression was lost upon knockdown of *dmrt2* in sLRO cells. Histological sections revealed that the endodermal layer, which is located at a distance to sLRO cells in control embryos (Supplemental Figure S2A), is shifted and located next to notochordal cLRO cells in *dmrt2* morphants (Supplemental Figure S2B). Taken together, these data strongly suggest that the presence, but not apical constriction, of sLRO cells depends on *dmrt2* activity.

### Dmrt2 is Required for sLRO Specification at Early Gastrula Stages

Next, we asked whether Dmrt2 function during paraxial patterning or myogenesis caused the absence of *nodal1*-expressing sLRO cells in neurula stages. In order to address such a connection during *Xenopus* LR development, we first analyzed the two myogenic marker genes *tbx6* and *myf5* in *dmrt2* morphants. The genes were chosen as 1) *tbx6* knockout mice display LRO defects and 2) murine *myf5* is a direct transcriptional target of Dmrt2 (Hadjantonakis et al., 2008; Sato et al., 2010; Concepcion et al., 2018). At neurula stages, both genes were expressed in presomitic mesoderm and importantly in sLRO cells (Supplemental Figures S3A, B), strongly suggesting a connection between myogenic pathways and sLRO morphogenesis. We thus analyzed *tbx6* and *myf5* expression in gastrula embryos, which had been unilaterally injected with *dmrt2* MO. Both genes were strongly downregulated when *dmrt2* function was inhibited (Figures 2E–L). The reintroduction of *dmrt2* mRNA statistically significantly restored *tbx6* expression (Figure 2H), further underscoring MO specificity. These results showed that Dmrt2 acts upstream of the myogenic transcription factors *tbx6* and *myf5,* the latter being in accordance with published data in mice (Sato et al., 2010).

**FIGURE 3:**
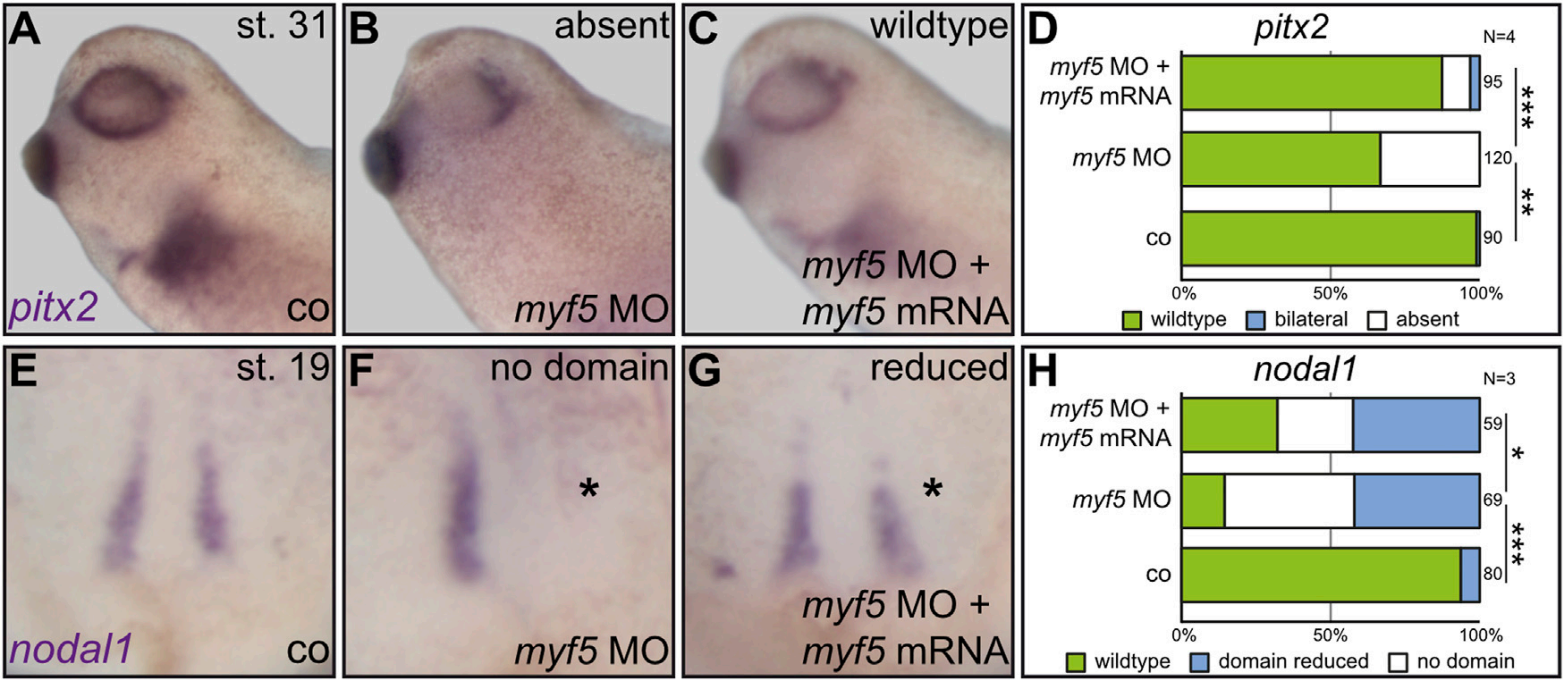
The myogenic transcription factor Myf5 is required for LR asymmetry. In order to connect paraxial patterning to LR development, a *myf5* knockdown was conducted in the left sLRO lineage and assayed for *pitx2* asymmetry at tadpole **(A–D)** or *nodal1* at neurula stages **(E–H)**. In contrast to controls [co;**(A)**], *myf5* morphants lost left *pitx2* expression **(B)**, which was regained by the introduction of *myf5* rescue mRNA **(C)**. Statistical analysis demonstrates the specificity of results **(D)**. At late neurula, *nodal1* expression was lost or reduced by *myf5* knockdown **(F)** which was partially rescued by co-injecting a *myf5* rescue mRNA **(G)**. Statistical analysis is depicted in **(H)**. Asterisks mark injected sides. Numbers in **(D,H)** represent analyzed specimens. N represents the number of independent experiments. Statistical analyses were done with one-sided Pearson’s chi-square test. * significant *p* < 0.05; ** highly significant *p* < 0.01; *** very highly significant *p* < 0.001.

Together, the above results showed that Dmrt2 regulates both somitogenic and sLRO genes, suggesting a novel functional link between somitogenesis and the processing of LR cues. If patterning of the paraxial mesoderm is linked to LR asymmetry, the loss of function of myogenic key genes should impact laterality. We, therefore, turned to manipulate *myf5* (Pownall et al., 2002), which, unlike *tbx6,* has not been implicated in LR axis formation. Tbx6 was shown to transcriptionally activate *myf5* (Li et al., 2006), rendering *myf5* an ideal downstream target for the loss-of-function experiments. A translation blocking MO was used to analyze the role of *myf5* during LR axis formation. At st. 31, control embryos expressed *pitx2* on the left side (Figures 3A, D). Right-sided *myf5* MO injections had no effect on *pitx2* asymmetry (data not shown). However, applying *myf5* MO to the left sLRO lineage prevented *pitx2* induction (Figures 3B, D). The loss of *pitx2* asymmetry was specific, as left *pitx2* expression was restored in morphants co-injected with *myf5* rescue mRNA (Figures 3C, D). Next, we analyzed sLRO specification by detecting *nodal1* mRNA (Figures 3E–H). Left-sided *myf5* knockdown either impeded *nodal1* expression entirely or substantially reduced its domain (Figures 3F, H). In addition, sLRO cells were not present in *myf5* morphants as visualized by the lack of *myod1* expression (Supplemental Figure S2). The reintroduction of *myf5* mRNA reduced the severity of the loss-of-function phenotype, suggesting MO specificity (Figures 3G, H). This demonstrates a crucial role for the myogenic transcription factor *myf5* in LR axis determination, as it specifies the sensory cells of a functional LRO.

**FIGURE 4:**
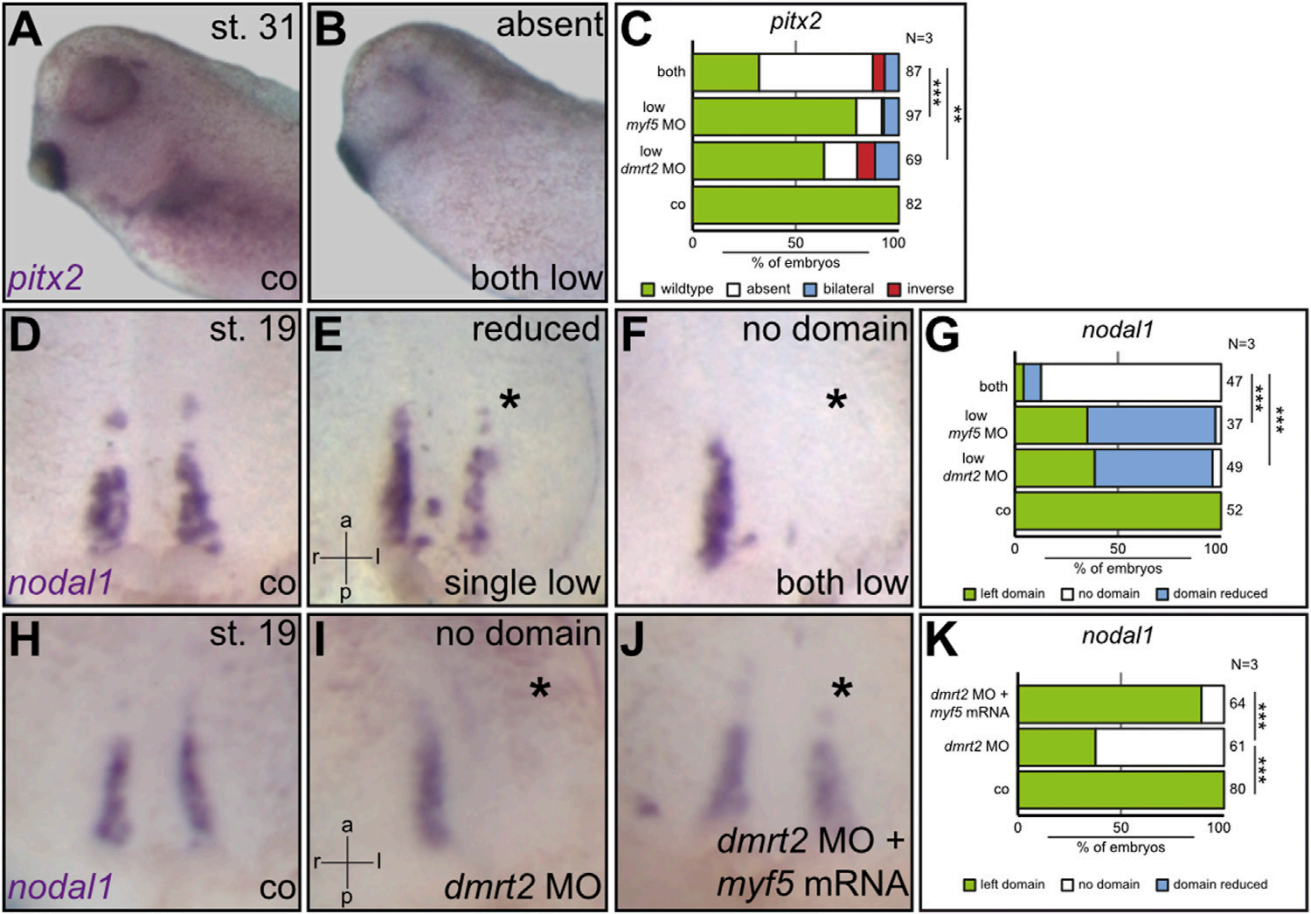
Myf5 specifies sLRO cells downstream of Dmrt2. Using suboptimal *dmrt2* MO and *myf5* MO dosages, the cooperation of both transcription factors was demonstrated at the level of *pitx2* asymmetry **(A–D)** and *nodal1* expression in sLRO cells **(D–G)**. The combination of both MOs resulted in an efficient loss of *pitx2* and *nodal1* expression. Note that individual MO injections had a weak impact on *nodal1* in sLRO cells. Co-injecting *myf5* mRNA rescued *nodal1* expression in *dmrt2* morphants **(H–K)**. Asterisks mark injected sides. Numbers in **(C,G,K)** represent analyzed specimens. N represents the number of independent experiments. Statistical analyses were done with one-sided Pearson’s chi-square test. ** highly significant *p* < 0.01; *** very highly significant *p* < 0.001. a = anterior; co = control; l = left; p = posterior; r = right.

The loss of function of *myf5* ultimately phenocopied the *dmrt2* loss of function, strongly suggesting that both act in the same pathway. To test whether both genes co-operate, suboptimal *dmrt2* MO and *myf5* MO doses were injected either individually or together into the sLRO lineage. Compared to control embryos, which expressed *pitx2* exclusively on the left side (Figure 4A), the individual injection of each MO at a low dose affected LR development in 20–30% of embryos (Figure 4C). However, in embryos that received a combination of both MOs at a low dose, *pitx2* expression was altered in the 75% of cases (Figures 4B, C), suggesting functional cooperation of *myf5* and *dmrt2* in LR determination. Formally, the genes could interfere with LR development individually at different stages or in different tissues. To demonstrate that both, *dmrt2* and *myf5*, act together in the same process, i.e. the specification of sLRO cells, *nodal1* transcription was analyzed at neurula stages using the same experimental setup as described above. Compared to controls (Figures 4D, G), individual injection of suboptimal doses of *dmrt2* MO or *myf5* MO mildly reduced the *nodal1* expression domain in about 60% of specimens (Figures 4E, G). In contrast, co-injecting low concentrations of *dmrt2* MO and *myf5* MO entirely prevented *nodal1* expression in 80% of specimens (Figures 4F, G). These results strongly argue that Dmrt2 and Myf5 jointly specify sLRO tissue. Next, we investigated whether this functional relationship was epistatic. The strong effect of *dmrt2* loss of function on *myf5* expression during gastrula stages suggests that *myf5* acts downstream of *dmrt2.* Indeed, the loss of *nodal1* expression in *dmrt2* morphants was very efficiently rescued by co-injecting *myf5* mRNA (Figures 4H–K). The frequency of restored *nodal1* transcription almost reached WT levels, demonstrating the sequential order of gene activities. Together, these results show that *dmrt2* governs both cLRO morphogenesis in the axial midline as well as paraxial mesoderm patterning. We identify the joint specification of sLRO and somitic cells as a prerequisite for LR axis specification.

## Discussion

### Bilateral Symmetry vs. LR Asymmetry—A Contradiction?

The aim of this study was to reveal a functional interaction between LR axis specification and paraxial patterning/somitogenesis. At first glance, it appears that these two processes are mutually exclusive in vertebrate embryos and must thus occur independently of each other: the perfect symmetry of somitogenesis which lays the ground for the symmetric formation of vertebrae and ribs, must not be disturbed by the asymmetry created along the LR axis.

During somitogenesis, a complex gene regulatory network that includes oscillating gene expression is orchestrated to ensure perfectly bilaterally symmetric development. Asymmetries in this context could result in nonfunctional musculature and skeletal defects, threatening the survival of the embryo. On the other hand, a highly complex mode of symmetry breakage, generated by a cilia-driven flow of extracellular fluids, is translated into the asymmetric release of the very potent morphogen Nodal. Nodal transfers leftness into the LPM and therefore could broadly impact various neighboring tissues along the left anterior-posterior axis. Indeed, several reports showed that Nodal interferes with the left somitic clock, i.e., the oscillatory gene expression module, in mice and chicks. Retinoic acid (RA) is thought to prevent such interference by shielding left-sided somites from Nodal-induced signal transduction (Vermot and Pourquié, 2005; Sirbu and Duester, 2006; Brend and Holley, 2009; Grimes, 2019). However, RA-mediated protection acts much later than the factors that we identify here, showing that both reflect distinct processes. Interestingly, the loss of *dmrt2* in fish desynchronized the somitic clock and led to LR defects, underscoring a molecular link between both processes (Saúde et al., 2005; Liu et al., 2009). Although we have not analyzed somite segmentation in *Xenopus*, we have identified a potential mechanism in which genes required for somitogenesis also act on LRO specification and morphogenesis.

### The Connection Between the sLRO and Somitogenesis

In *Xenopus*, cell labeling experiments have demonstrated that sLRO cells are fated to become somitic tissue. More specifically, after flow sensing, sLRO cells ingress into the somites and differentiate into the horizontal myoseptum which divides the somite into dorsal and ventral regions (Shook et al., 2004). On the molecular level, we confirmed these observations by showing that the myogenic marker genes *myod1*, *tbx6,* and *myf5* are expressed in the sensory part of the LRO (Schweickert et al., 2010; Schneider et al., 2019 and this work). Functionally, we demonstrated a requirement of Dmrt2 and Myf5 for sLRO specification/morphogenesis. The role of *myf5* during LR development was particularly unanticipated because the involvement of a *bona fide* myogenic transcription factor in LR axis formation was not reported so far. Therefore, we conclude that in *Xenopus*, paraxial patterning, i.e. somitogenesis, is functionally linked to symmetry breakage.

### Cell-Autonomous vs. Non-Cell-Autonomous Functions of Dmrt2

We found that Dmrt2 is required for SM specification and paraxial patterning at gastrula stages. These early events are thus essential to establish a leftward flow driven by motile cilia on the cLRO and its subsequent left-sided sensing in somitic sLRO cells. How flow is perceived remains an open question. But is Dmrt2 acting in a cell-autonomous or non-cell-autonomous manner, i.e. in the SM or in the underlying DM? We detected *dmrt2* transcripts in both cell layers (Supplemental Figures S1A, B) which did not allow for a differentiation between both modes. However, since *foxj1* is exclusively expressed in SM cells (Stubbs et al., 2008; Beyer et al., 2012) and *foxj1* is a transcriptional target of Dmrt2 in fish (Pinto et al., 2018), a cell-autonomous Dmrt2 activity to induce *foxj1* expression seems plausible. Importantly, this likely applies to the axial part of the SM, the cLRO precursor cells, but not to lateral SM cells which are fated to develop into sLRO tissue. Our dissection approach at gastrula stages did not discriminate between axial and lateral SM or between axial (notochordal) and lateral (presomitic) DM. As *myf5* expression is restricted to the lateral deep mesodermal layer (cf. Supplemental Figure S4) and because Myf5 acts epistatic to Dmrt2 during specification of *nodal1*-positive sLRO cells (Figure 4), a non-cell-autonomous activity for Dmrt2 seems to be plausible, too.

We have recently reported that Fgf signaling also plays a dual role during LRO formation (Schneider et al., 2019). Blocking Fgf signaling prior to gastrulation diminished *foxj1* expression in gastrula embryos (Schneider et al., 2019), which is indicative of impaired SM specification and consequently loss of LRO cilia and loss of leftward flow (Stubbs et al., 2008; Beyer et al., 2012). This function is probably conserved, as LRO morphogenesis in mice and fish depends on Fgf signaling as well (Hong and Dawid, 2009; Sudheer et al., 2016). When Fgf signaling was blocked from mid-gastrula stages onward, *foxj1* expression and LRO ciliation were not affected, but induction of the left-sided Nodal cascade failed due to a loss of sLRO cells (Schneider et al., 2019).

**FIGURE 5:**
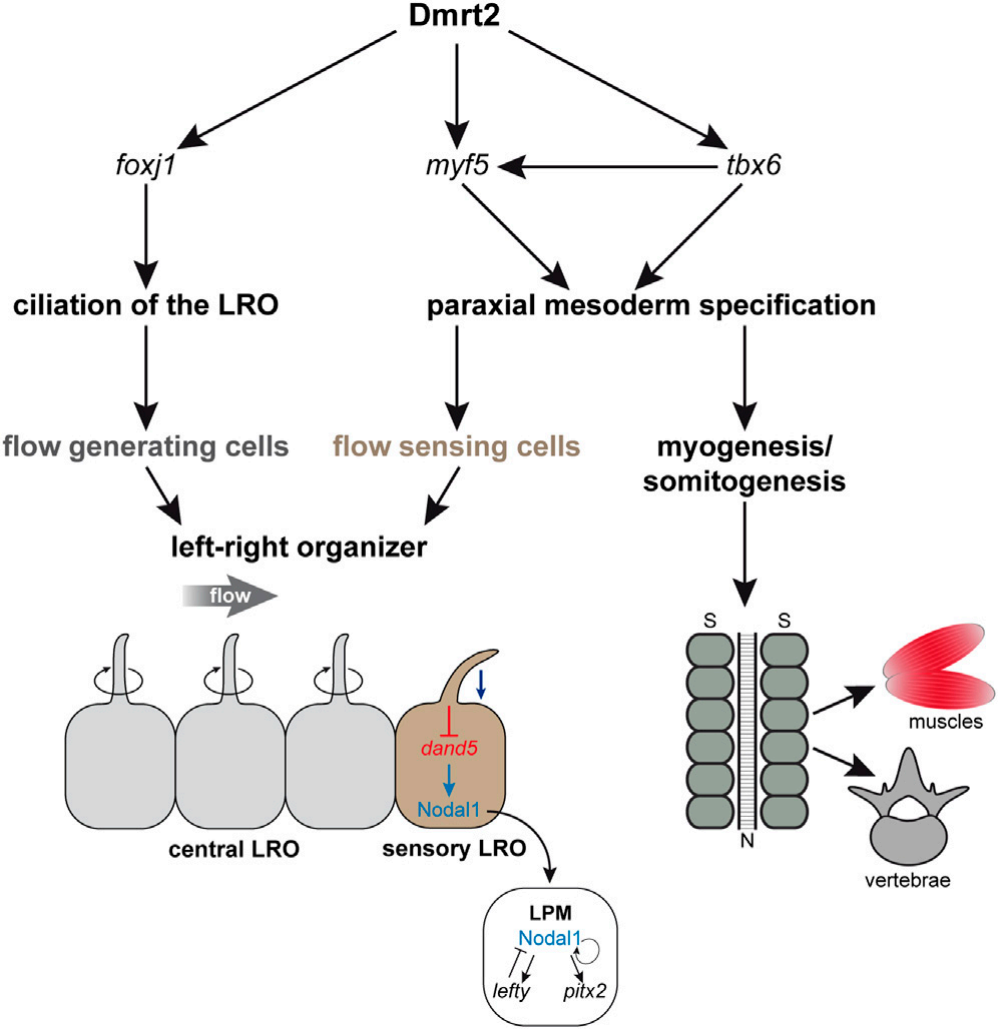
Dmrt2 regulates symmetry breakage and early somitogenesis in *Xenopus laevis*. Dmrt2 intervenes with two processes during symmetry breakage. It specifies the LRO by inducing *foxj1* in the SM that gives rise to the ciliated epithelium, which generates a leftward flow of extracellular fluid (left panel). Simultaneously, Dmrt2 activates *tbx6* and *myf5* expression in the early gastrula embryo, leading to the specification of the paraxial mesoderm (right panel), which later differentiates into muscles and vertebrae. In addition, the somatic functions of Dmrt2 and Myf5 are required for sensory LRO morphogenesis and thus for Nodal-cascade induction in the left LPM. N = notochord; S = somites.

*dmrt2* LOF also leads to a loss of sLRO cells. However, Dmrt2 functions in early gastrulae, i.e. substantially earlier than the time point at which the inhibition of Fgf signaling induces loss of sLRO cells. Interestingly, *myf5*, which is absent in *dmrt2* morphants, induces the somitic expression of Fgf4 and Fgf6 in mice. Via this route, it may provide a secondary Fgf signal for sLRO morphogenesis (Grass et al., 1996; Fraidenraich et al., 2000). As only SM cells develop into the sLRO and since *myf5* mRNA is only present in the DM that does not contribute to the LRO, it is still unclear how Myf5 is able to regulate sLRO formation. Together with published data, our observations suggest that Myf5 in the DM influences specification of the SM in a non-cell-autonomous manner, potentially via secreted Fgf ligands. The existence of two temporally distinct Fgf pathways is in agreement with published work on the role of Fgf during gastrulation. Early Fgf signaling is transduced by the MAPK pathway, whereas the late Fgf signal uses calcium as a second messenger (Nutt et al., 2001; Sivak et al., 2005). It remains to be seen whether Fgf ligands induced by Myf5 trigger the Fgf/Ca2^+^ pathway for sLRO specification and/or morphogenesis. In a hierarchical model, Dmrt2, potentially induced by an early Fgf signal, induces *foxj1* in the LRO precursor tissue, which is required for ciliogenesis and for setting up a leftward flow. In parallel, Dmrt2 induces the myogenic genes *tbx6* and *myf5*. Myf5, possibly via a second phase of Fgf signaling, induces sLRO specification and morphogenesis. In this dual setting, Dmrt2 represents a crucial factor for LR determination in *Xenopus laevis* (Figure 5).

### Evolutionary Aspects of Dmrt2 Function in LR and Somitogenesis

To what extent is the role of *dmrt2* evolutionarily conserved and thus transferable to other vertebrate species? It is most likely that Dmrt2-dependent LRO morphogenesis is conserved in fish. This notion is supported by three recent publications: 1) zebrafish with diminished *dmrt2* levels develop LR and somitogenesis defects (Saúde et al., 2005; Liu et al., 2009); 2) the zebrafish LRO expresses *dmrt2* (Lourenço et al., 2010), and 3) in zebrafish, the master control gene for the biogenesis of motile cilia *foxj1*, is a transcriptional target of Dmrt2 (Pinto et al., 2018). Surprisingly, *dmrt2* knockout mice do not exhibit LR defects, suggesting that mouse LRO morphogenesis is independent of Dmrt2 (Lourenço et al., 2010). A potential interpretation of this finding is that mammalian Dmrt2 has lost its LR function during evolution, which is therefore found in lower vertebrates only (see below). Cell lineage analysis showed that murine LRO (node, posterior notochord) cells, have notochord identity (Wilson and Beddington, 1996; Kinder et al., 2001; Yamanaka et al., 2007; Wang and Ware, 2009; Babu and Roy, 2013). In addition, sLRO (crown cell)-specific transgenes, which are widely used (e.g. NDE-lacZ; Brennan et al., 2002; Krebs et al., 2003), have not been reported to mark somitic cells. Together, this renders evolutionary conservation of the cell lineage of the mouse and *Xenopus* LRO implausible. This might explain the lack of an LR phenotype in the *dmrt2* knockout mouse (Saúde et al., 2005).

However, a link of LR asymmetry with somitogenesis appears to be conserved in other vertebrates. The human Klippel-Feil syndrome (KFS) is characterized by segmentation defects of the vertebrae, pointing to impaired embryonic somitogenesis. Intriguingly, several KFS case reports describe the concomitant occurrence of laterality defects, suggesting that somitogenesis and LR are linked in humans. Interestingly, mutations in the human *GDF3* gene have been found to be causative of KFS (Jalil et al., 2008; Chacón-Camacho et al., 2012; Futane and Salunke, 2013; Karaca et al., 2015; Abdali et al., 2021). Therefore, GDF3 could directly connect KFS clinical pictures to its well-established function during laterality determination. Unfortunately, the genetic basis of KFS patients showing *situs inversus* or heterotaxia has not been mapped in most cases and needs further experimental validations.

In contrast to mice, labeling of the fish LRO precursor cells showed a notochordal and a somitic cell fate (Melby et al., 1996), indicating homology to *Xenopus*. However, the specific whereabouts of *dand5*/*nodal* positive sLRO cells have not been addressed so far. In *Medaka, nodal* was detected in presomitic mesoderm at the early LRO, prior to flow and *dand5* asymmetry (Hojo et al., 2007). Based on the functional similarities of frog and fish *dmrt2,* a somitic fate in both species seems plausible. This argument is strongly supported by a recent report. In zebrafish, it was demonstrated that a *dand5* promotor-driven EGFP transgene marked LRO cells, which at later stages were found to be integrated into the axial and presomitic mesoderm (Ikeda et al., 2022). Interestingly, studies in sauropsida such as turtles, geckos, and the chick identified bilateral *nodal* expression domains at the embryonic midline that have somitic cell fates (Otto et al., 2014; Kajikawa et al., 2020). This is notable because this vertebrate clade induces asymmetry by an as yet unidentified mechanism. This unknown process triggers downregulation of right-sided paraxial *nodal*, resulting in Nodal cascade induction only in the left LPM. In chick embryos, two extracellular inhibitors of the Cerberus family, caronte and cerberus itself are initially expressed in the presomitic mesoderm and were shown to be required for chick LR development (King and Brown, 1999; Esteban et al., 1999; Yokouchi et al., 1999; Yu et al., 2008; Katsu et al., 2012). From an evolutionary point of view, co-expression of Nodal and a Cerberus-related inhibitor seems to be a module that is conserved and active in LR determination of all vertebrates. This notion is further underscored by the development of the cephalochordate *Branchiostoma*, an animal that exhibits LR asymmetries of all organs and tissues, including the somites. Like in the frog, a cilia-driven leftward flow downregulates *dand5* in *Branchiostoma*, which allows activation of a left-sided Nodal cascade. Unlike vertebrates, both processes, flow-dependent *dand5* inhibition and Nodal cascade propagation are restricted to only one tissue, the presomitic mesoderm. In consequence, asymmetric gene expression induces asymmetric differentiation of somites and other tissues during embryogenesis (Blum et al., 2014a; Soukup et al., 2015; Li et al., 2017; Soukup, 2017; Zhu et al., 2019). We, therefore, postulate that a “Nodal/Cerberus-like inhibitor” module is conserved among vertebrates, although the modes of symmetry breakage change during evolution. It remains an open question whether our findings in the frog also apply to other vertebrate species. Taken together, we showed that paraxial mesodermal patterning specifies the sensory part of the LRO, thereby conjoining two embryonic processes that appear mutually exclusive at first glance.

## Materials and Methods

### Experimental Animal

*Xenopus laevis* were obtained from Nasco (901 Janesville Avenue PO Box 901 Fort Atkinson) and were treated in accordance with German Regulations and laws approved by the Regional Government Stuttgart (A379/12 Zo, “Molekulare Embryologie”, V340/17 ZO and V349/18 ZO, “*Xenopus* Embryonen in der Forschung”).

### Plasmids and mRNA Synthesis

A *dmrt2* probe (1,481 bp) for WMISH was amplified by RT-PCR using a 5′UTR forward primer 5′TCC​CAC​CAC​TAA​GGG​AAC​TG3′ and fourth exon reverse primer 5′TTTTCAAGATG TGCCTGCTG3′ and cloned into the pGEMT-easy vector. For rescue experiments, full-length *dmrt2* (corresponding to NM_001096256.1) was amplified by RT-PCR and cloned into the pCS2+ vector. The following primers were used: Forward 5′ATCGGGATCCTTAGAAATGTATGAAATGAAAGCGCCTGCTGCCCCATCCTCTTCCTCGT3'; Reverse 5′ATCCATCGATGTTACTGACTAGAACGCTTGACTGTTGT TGAGGG3'.

Full-length myf5 in pBSK+ was a gift from V. Gawantka and C. Niehrs (corresponds to NM_001101779.2). For the gain of function experiments, *myf5* was cloned into pCS^2+^ by restriction digest using EcoRI. A *myf5* rescue construct was generated by PCR using forward 5′ATATCGATAT GGA​AAT​GGT​TGA​CAG​TTG​TCA​CTT​C3′ and reverse 5′ATG​GAA​ATG​GTT​GAC​AGT​TGT CACTTC3′ oligonucleotides.

For mRNAs synthesis, pCS^2+^ expression vectors were linearized by SacII (*dmrt2*) or NotI (*myf5*) and transcribed using the Invitrogen mMessage sp6 kit according to user instructions.

### Microinjection and Morpholino Sequences

A volume of 4 ml was microinjected into the left dorsal marginal region of 4 and 8-cell stage embryos. Bilateral injections were performed for flow analysis. Antisense morpholinos were provided by GeneTools. *dmrt2* MO 5′ TGC​CTT​CAT​CTC​GTA​CAT​CTC​CAG​C 3′ and *myf5* MO 5′ ACC​ATC​TCC​ATT​CTG​AAT​AGT​GCT​G 3′were injected at a concentration of 1pMol/embryo. *dmrt2* and *myf5* mRNAs were applied at a concentration of 50–100 ng/μl or 50–60 ng/μl, respectively.

### RT-PCR and qPCR Analysis

Superficial and deep mesodermal tissue of stage 10.5 embryos were manually dissected and separated in CMFM buffer (Calcium Magnesium Free Medium, 88 mM NaCl, 1 mM KCl, 2.4 mM NaHCO_3_, 7.5 mM Tris (pH 7,6); (Sargent et al., 1986). RNA was isolated by phenol-chloroform extraction. For cDNA synthesis and qPCR analysis, the Promega Kit GoTaq 2-Step RT-qPCR System (A6010) was used according to user instructions. Real-time quantitative PCR (RT-qPCR) was carried out in a 96-well plate on the Roche LightCycler System 96. Each sample was conducted in triplicates (technical replicates) and relative expression was calculated by ΔΔCT-method.

Primers used for conventional RT-PCR: *dmrt2L_x1* forward 5′TGG​ACT​TTT​CTT​ACC​TAA​CCG​C3′ and *dmrt2L_x1* reverse 5′TGA​CTC​CTT​TCC​TAA​GAA​GCA​GT3'. The primers *odcL* forward 5′TGC​AGA​GCC​TGG​GAG​ATA​CT3′ and *odcL* reverse: 5′GGC​AGC​AGT​ACA​GAC​AGC​AG3′ served as positive control. Primers for qPCR: *dmrt2L_x1* forward 5′CAAAGCCCAGCATC ACAGAG3′ and *dmrt2L_x1* reverse 5′TGG​TCC​CCA​GGT​AAG​AAT​CAG3'. Reference genes for qPCR (Mughal et al., 2018): *sub1L* forward 5′AGC​AGG​AGA​AAT​GAA​GCC​AGG3′, *sub1L* reverse 5′CCG​ACA​TCT​GCT​CCT​TCA​GT3′ and *slc35b1L forward* 5′CGC​ATT​TCC​AAA​CAG​GCT​CC3′, *slc35b1L* reverse 5′CAA​GAA​GTC​CCA​GAG​CTC​GC3'.

### RNA *In Situ* Hybridization

SP6 or T7 RNA polymerase (Promega) was used to synthesize Digoxigenin-labeled (Roche) RNA probes from linearized plasmids. *tbx6* probe was kindly provided by Hideho Uchiyama. MEMFA was used to fix embryos and processed them following standard protocols. Whole-mount *in situ* hybridization (WMISH) was carried out according to Belo et al., 1997.

### Leftward-Fluid Flow Analysis and Immunofluorescence

Flow analysis was carried out as described (Schweickert et al., 2007; Tingler et al., 2018).

For immunofluorescence, the monoclonal mouse anti-acetylated *α*-tubulin antibody (1:700; T6798 Sigma) and a secondary anti-mouse antibody (1:1,000; c2181 Sigma) were used and conducted as described (Tingler et al., 2018).

### Statistics

Comparisons of altered marker gene expression (*pitx2*, *foxj1*, *myf5*, *tbx6*) were statistically analyzed using one-sided Pearson’s chi-square test in statistical R. Statistical relevance of flow directionality and velocity as well as cilia length was calculated by the Wilcoxon-Match-Pair test (statistical R-3.0.1).

## Data Availability

The original contributions presented in the study are included in the article/Supplementary Material; further inquiries can be directed to the corresponding author.
