# Supplementary material for: dmrt2 and myf5 Link Early Somitogenesis to Left-Right Axis Determination in Xenopus laevis
Source: Front Cell Dev Biol. 2022 Jun 23;10:858272. doi: 10.3389/fcell.2022.858272 (PMC9260042; doi:10.3389/fcell.2022.858272)
Supplement: Supplementary file 1 [file DataSheet1.docx]

Supplementary Material

**
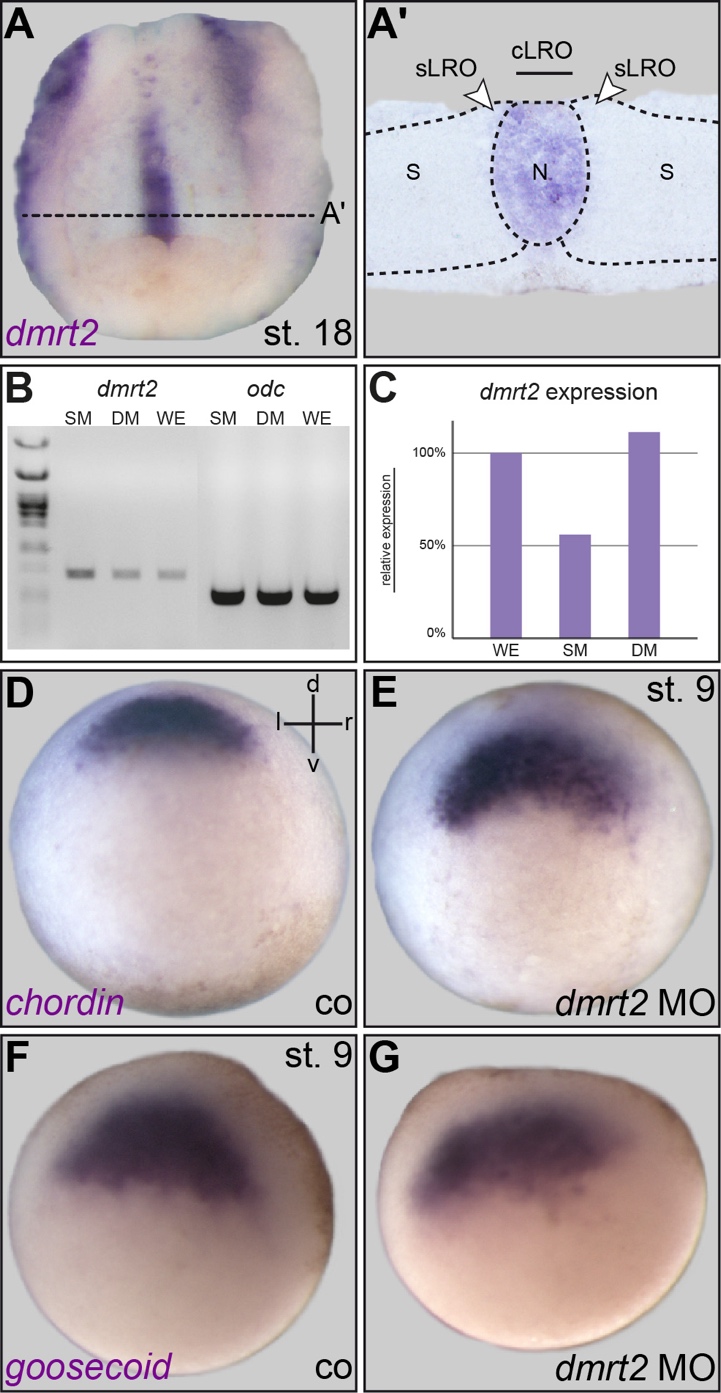
**

**Supplemental Figure 1: *dmrt2* expression in cLRO cells / dissected SM/DM tissues and organizer gene expression in *dmrt2* morphants.**WMISH and subsequent histological sections of neurula stage (st.) embryos revealed *dmrt2* transcription in cLRO cells and the notochord (N). Note that lateral sLRO cells and somites (S) lack *dmrt2* expression (**A,** white arrowheads in **A'**). Detection of *dmrt2* transcripts by RT-PCR (**B**) and RT-qPCR (**C**) in dissected superficial mesoderm (SM), underlying deep mesoderm (DM) and whole embryo (WE) at stage (st.) 10.5. *odc* served as loading control in **A**. Spemann Organizer marker genes *chordin* (**D, E)** and *goosecoid* (**F, G** ) were not perturbed in *dmrt2* morphants **(**. a=anterior; d=dorsal; l=left; r=right; co=control; cLRO=central left-right organizer; sLRO= sensory left-right organizer.

**
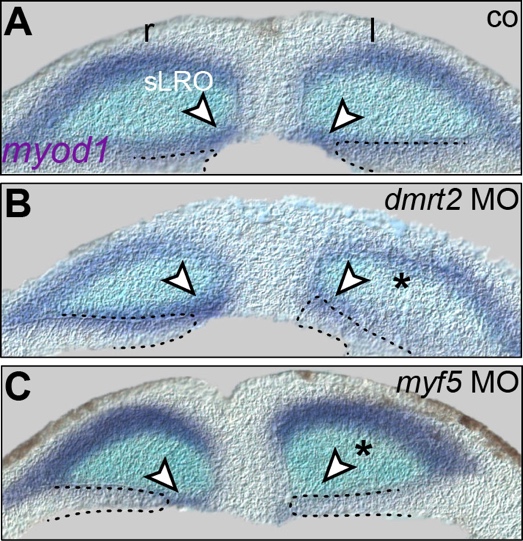
**

**Supplemental Figure 2: sLRO cells are lost in *dmrt2* and *myf5* morphant neurulae.**

Histological sections showed *myod1* expression in control **(A)**, unilaterally *dmrt2* MO (**B**) or *myf5* MO (**C**) injected embryos at neurula stage (st. 18). In controls (co) and in the non-injected side of morphants, *myod1* positive cells border to the extra cellular space of the archenteron sLRO. Following *dmrt2* or *myf5* knockdown, *myod1* expression is restricted to deep cells, which are covered by the endodermal cell layer (dashed line). White arrow heads point to the position of sLRO cells. Asterisks mark injected sides; l=left; r=right; co=control; sLRO= sensory left-right organizer.

**
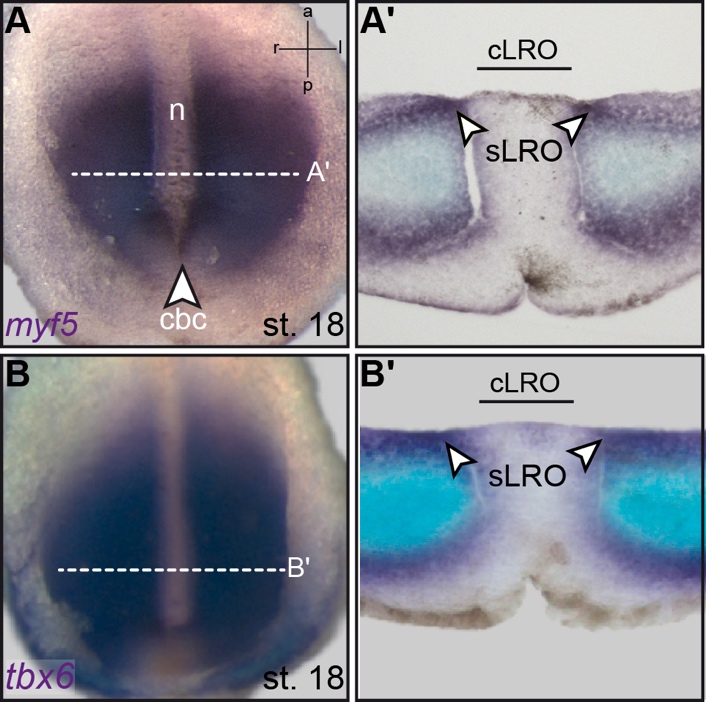
**

**Supplemental Figure 3: *myf5* and *tbx6* are expressed in sLRO cells.**

Both, *myf5* (**A**) and *tbx6* (**B**), are expressed in the posterior presomitic mesoderm in neurula stages (st. 18). Transversal sectioning demonstrated that *myf5* (**A’**) and *tbx6* (**B’**) positive signals were present in the presomitic mesoderm, including sLRO cells. White arrow heads point to the position of sLRO cells. Plain of section is indicated in **(A, B)** by a dashed line. cbc, circular blastoporal collar; sLRO, sensory left-right organizer; cLRO, central left-right organizer.

**
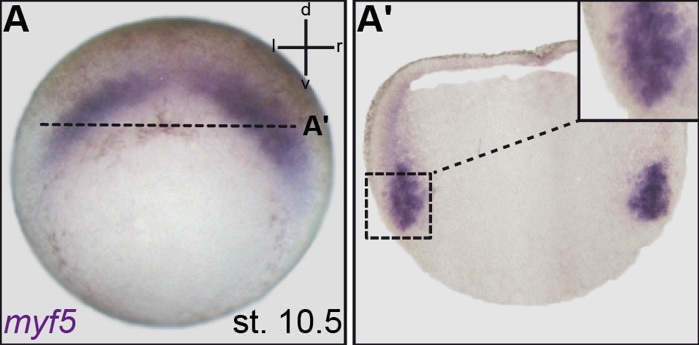
**

**Supplemental Figure 4: *myf5* expression in early gastrula embryos is restricted to deep cells.** *myf5* staining of a stage (st.) 10.5 gastrula, shown in a dorso-vegetal view **(A)**. Transversal section revealed that *myf5* mRNA is present only in deep mesodermal cell populations (DM) but not in the superficial layer **(A')**. Dashed line in **(A)** indicated the plain of section.
